# Formation of a cytoplasmic salt bridge network in the matrix state is a fundamental step in the transport mechanism of the mitochondrial ADP/ATP carrier

**DOI:** 10.1016/j.bbabio.2015.09.013

**Published:** 2016-01

**Authors:** Martin S. King, Matthew Kerr, Paul G. Crichton, Roger Springett, Edmund R.S. Kunji

**Affiliations:** Medical Research Council Mitochondrial Biology Unit, Cambridge Biomedical Campus, Wellcome Trust/MRC Building, Hills Road, Cambridge, CB2 0XY, UK

**Keywords:** AAC, ADP/ATP carrier from *Myceliophthora thermophila*, CATR, carboxyatractyloside, BKA, bongkrekic acid, CPM, N-[4-(7-diethylamino-4-methyl-3-coumarinyl)-phenyl]-maleimide, Adenine nucleotide translocase, Membrane protein, Substrate exchange, Thermostability, Transport protein

## Abstract

Mitochondrial ADP/ATP carriers catalyze the equimolar exchange of ADP and ATP across the mitochondrial inner membrane. Structurally, they consist of three homologous domains with a single substrate binding site. They alternate between a cytoplasmic and matrix state in which the binding site is accessible to these compartments for binding of ADP or ATP. It has been proposed that cycling between states occurs by disruption and formation of a matrix and cytoplasmic salt bridge network in an alternating way, but formation of the latter has not been shown experimentally. Here, we show that state-dependent formation of the cytoplasmic salt bridge network can be demonstrated by measuring the effect of mutations on the thermal stability of detergent-solubilized carriers locked in a specific state. For this purpose, mutations were made to increase or decrease the overall interaction energy of the cytoplasmic network. When locked in the cytoplasmic state by the inhibitor carboxyatractyloside, the thermostabilities of the mutant and wild-type carriers were similar, but when locked in the matrix state by the inhibitor bongkrekic acid, they correlated with the predicted interaction energy of the cytoplasmic network, demonstrating its formation. Changing the interaction energy of the cytoplasmic network also had a profound effect on the kinetics of transport, indicating that formation of the network is a key step in the transport cycle. These results are consistent with a unique alternating access mechanism that involves the simultaneous rotation of the three domains around a central translocation pathway.

## Highlights

• Mitochondrial ADP/ATP carriers alternate between the matrix and cytoplasmic state.
• Matrix and cytoplasmic salt bridge networks regulate access to central binding site.
• Thermostability assays are used to probe state-dependent interactions in carriers.
• Cytoplasmic salt bridge network mutations only affect matrix state thermostability.
• Formation of the cytoplasmic network is a fundamental step in the transport cycle.

## Graphical abstract

**Figure:**
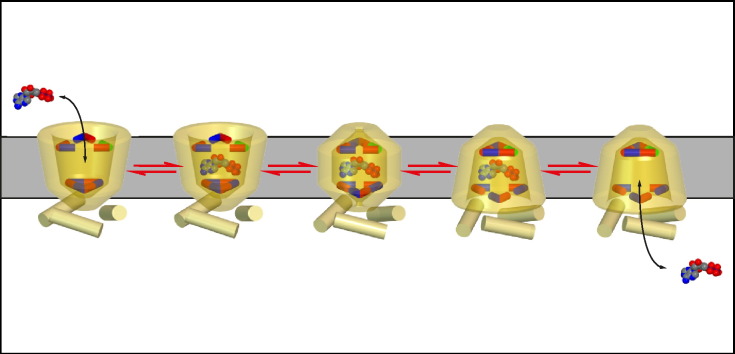

## 1 Introduction

**Fig. 1:**
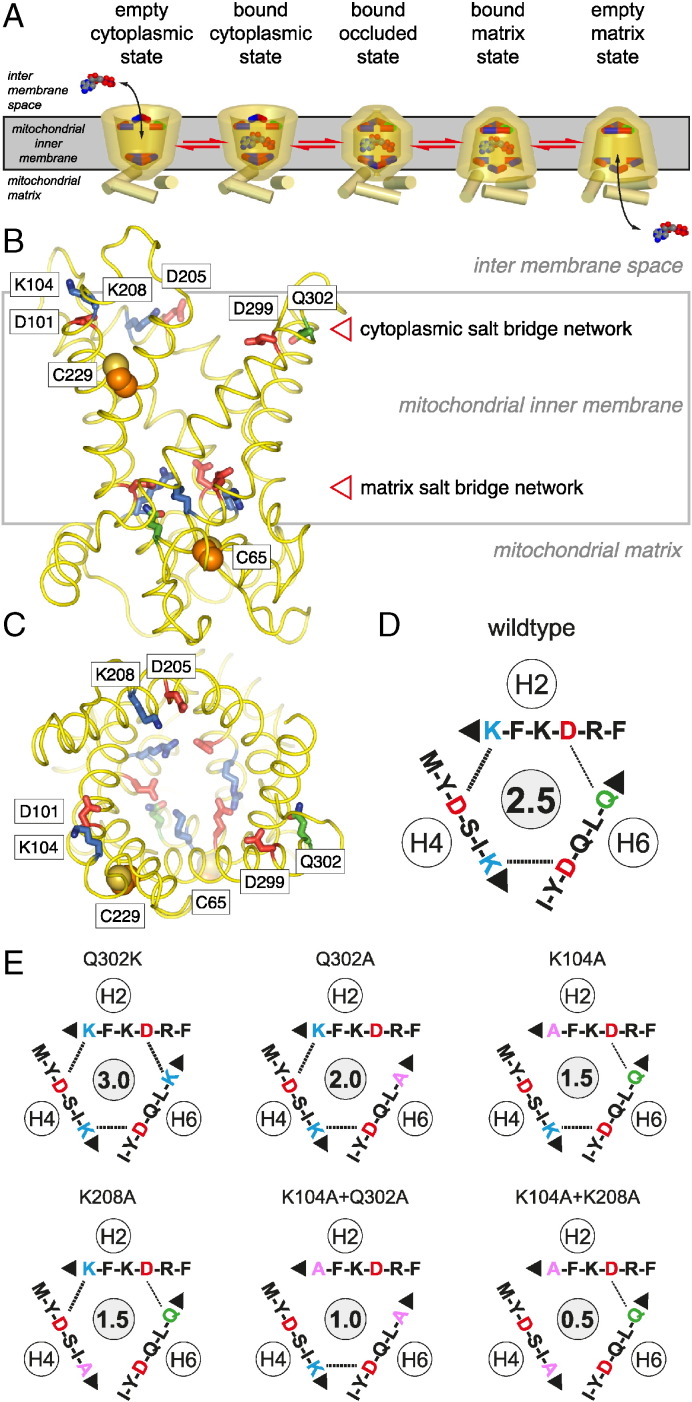
Transport states, architecture and cytoplasmic salt bridge network of the mitochondrial ADP/ATP carrier from *Myceliophthora thermophila*. (A) Reversible transport states of the carrier, consisting of an empty cytoplasmic state, bound cytoplasmic state, bound occluded state, bound matrix state, and empty matrix state. Disruption and formation of the cytoplasmic and matrix salt bridge networks, top and bottom respectively, have been proposed to be involved in the opening and closing of the carrier to either side of the membrane in an alternating way [16]. Membrane view (B) and cytoplasmic view (C) of the comparative homology model (yellow) generated with SwissModel [40] based on the related structure of Aac2p of *Saccharomyces cerevisiae* (PDB 4C9G) [21]. Acidic, basic and polar residues of the matrix and cytoplasmic salt bridge network are shown as red, blue and green sticks, respectively. The cysteine residues C65 and C229 are indicated by orange sphere representations. Schematic representations of the cytoplasmic salt bridge network of wild-type (D) and network mutants of AAC used in this study (E). The theoretical interaction energy is shown in the central gray circle, in which one point and half a point are assigned to each salt bridge and hydrogen bond, respectively [16].

Members of the mitochondrial carrier family transport chemically and structurally diverse compounds across the mitochondrial inner membrane with a common, yet unresolved, mechanism [1], [2], [3]. The mitochondrial ADP/ATP carrier (AAC), a member of the protein family, imports ADP into the mitochondrion for conversion to ATP and exports ATP synthesized by ATP synthase to the cytosol. The transport protein cycles between a cytoplasmic and matrix state in which the substrate-binding site is accessible to each of these compartments for binding of ADP and ATP (Fig. 1A) [4]. The specific inhibitors atractyloside and carboxyatractyloside (CATR) lock the transporter in an aborted cytoplasmic state, whereas bongkrekic acid (BKA) locks the carrier in an aborted matrix state [5], [6], [7], [8], [9]. These and other observations led to the formulation of the 'single binding centre gated pore mechanism' for mitochondrial carriers [4], which is essentially an alternating access mechanism [10].

Mitochondrial carriers consist of three homologous sequence repeats, each coding for two transmembrane α-helices [11]. A low-resolution projection structure of the yeast ADP/ATP carrier 3, inhibited by atractyloside, showed that the carrier was a monomeric three-fold pseudo-symmetrical α-helical bundle with a translocation pore through the center [12]. The first atomic structure of the bovine mitochondrial ADP/ATP carrier, isolated from native sources and inhibited by CATR, demonstrated that each of the three domains folds into two transmembrane α-helices that are connected by a short α-helix on the matrix side [13]. In the cytoplasmic state, the six transmembrane α-helices form a barrel around a central water-filled cavity, which is accessible to the mitochondrial intermembrane space and, *via* porins, to the cytosol [13]. By applying chemical and distance constraints [14], [15] and by symmetry analysis [16] a single substrate-binding site was identified in the central part of the cavity of the mitochondrial carriers. The same site was shown to bind ADP in the bovine ADP/ATP carrier in molecular dynamics simulations [17], [18]. The contact points of the substrate-binding site have been shown experimentally to be determinants of substrate specificity/selectivity [14], [15], [19].

The odd-numbered transmembrane α-helices H1, H3 and H5 on the matrix side of the carrier contain the signature motif of the mitochondrial carrier family Px[DE]xx[RK] [20]. The proline residues of this motif are found at sharp kinks of the L-shaped odd-numbered transmembrane α-helices, as they break the hydrogen bond arrangement [13], [21]. The charged residues form an inter-domain salt bridge network in the cytoplasmic state, closing the carrier on the matrix side [13]. Underneath the matrix network is a highly conserved glutamine residue that forms a brace between residues of the salt bridge between domain 1 and 3, increasing the interaction energy of the entire network [21]. The matrix network and glutamine braces are part of the matrix gate together with the C-terminal ends of the odd-numbered α-helices [13] and the h12 matrix loop [13], [21]. Another conserved motif [FY][DE]xx[RK] was identified on each of the even-numbered transmembrane α-helices [16]. These residues have been proposed to be part of the cytoplasmic gate. When the gate is closed in the matrix state the conserved aromatic residues may form a bulky hydrophobic layer [2], [21], whereas the charged residues of this motif might form a salt bridge network on the cytoplasmic side of the carrier [16]. We have previously shown by charge reversal mutagenesis that these charged residues interact as part of the transport cycle [21], but direct experimental evidence for the formation of the cytoplasmic network in the matrix state is not available.

For this study we have used the mitochondrial ADP/ATP carrier from *Myceliophthora thermophile* [22], a thermophilic fungus, which is stable in detergent solutions in the unliganded form. A series of site-directed mutations was created with the aim to increase or decrease the overall interaction energy of the proposed cytoplasmic network systematically. Mutant and wild-type AAC were purified, and a thermostability assay was used to assess the effect of the mutations on protein stability when the carriers were locked in either the cytoplasmic or the matrix state with CATR or BKA, respectively. The results show that specific interactions between residues of the cytoplasmic network form only when the carrier is in the matrix state. The mutations of the network also have a marked effect on the kinetics of transport, indicating that cytoplasmic network formation is a fundamental step in the transport cycle of the mitochondrial ADP/ATP carrier.

## 2 Materials and methods

### 2.1 Protein purification and thermostability assays

#### 2.1.1 Construction of yeast wild-type and mutant AAC expression strains

The codon-optimized gene of the mitochondrial ADP/ATP carrier from *Myceliophthora thermophila* was synthesized with an N-terminal eight histidine tag and Factor Xa cleavage site (Genscript) and cloned into the yeast expression vector pYES-PMIR2-AAC2 [23]. Site-directed mutations were introduced in the gene by using overlap-extension PCR [24] with KOD HotStart polymerase (Novagen). For the introduction of multiple mutations, several rounds of site-directed mutagenesis were performed. Expression vectors were transformed by electroporation into *Saccharomyces cerevisiae* strain WB12 (MATα ade2-1 trp1-1 ura3-1 can1-100 aac1::LEU2 aac2::HIS3) [25], which lacks functional Aac1p and Aac2p carriers. Transformants were selected initially on SC medium minus tryptophan plates, and then on YPG plates, confirming they expressed functional ADP/ATP carriers.

#### 2.1.2 Preparation of lipid for protein purification

Tetraoleoyl cardiolipin (18:1) dissolved in chloroform was purchased from Avanti Polar Lipids (Alabaster, Alabama). Typically, 100 mg of lipid was dispensed into a glass vial, and chloroform was removed by evaporation under a stream of nitrogen. Lipids were solubilized in 10% (*w*/*v*) dodecyl-maltoside by vortexing for 4 h at room temperature to give 10 mg ml^− 1^ lipid in a 10% detergent stock. The stocks were snap-frozen and stored in liquid nitrogen.

#### 2.1.3 Purification of wild-type and mutant AAC

For each wild-type and mutant AAC, a five-liter pre-culture was used to inoculate 100 l of YPG medium in an Applikon 140 Pilot System with an eZ controller. Cells were grown at 30 °C for 72 h, and harvested by centrifugation (4000 ×* g*, 20 min, 4 °C). Mitochondria were prepared with established methods [12], flash frozen in liquid nitrogen, and stored at − 80 °C until use. Isolated yeast mitochondria (250 mg total protein) were solubilized in 3% dodecyl-β-maltoside (Glycon Biochemicals GmbH) dissolved in a buffer consisting of 20 mM imidazole, 150 mM NaCl, 20 mM HEPES/NaOH, pH 8.0, and an EDTA-free complete protease inhibitor tablet (Roche Diagnostics Ltd.) by mixing at 4 °C for one hour. Particulate material was removed by ultracentrifugation (140,000 g, 45 min, 4 °C). The soluble fraction was loaded onto a Ni-Sepharose high performance column (Amersham Biosciences) at 1 ml min^− 1^ on an ÄKTAprime (GE Healthcare). The column was washed with 40 column volumes of buffer containing 20 mM HEPES/NaOH pH 8.0, 150 mM NaCl, 20 mM imidazole, 0.1% dodecyl-maltoside and 0.10 mg ml^− 1^ tetraoleoyl cardiolipin (18:1). The column material was washed with a further 20 column volumes of buffer B containing 20 mM HEPES/NaOH pH 8.0, 50 mM NaCl, 0.1% dodecyl-maltoside and 0.10 mg ml^− 1^ tetraoleoyl cardiolipin (18:1). For each preparation, the column material was resuspended with 400 μL buffer B, and transferred to a vial containing 5 mM CaCl_2_ and 75 μg Factor Xa (New England BioLabs), vortexed thoroughly, and incubated at 10 °C overnight. The following day, the cleaved protein was separated from the nickel sepharose media using filtration and centrifugation, the protein concentration was determined and adjusted to 3 mg ml^− 1^ with buffer B, and the sample was snap-frozen and stored in liquid nitrogen.

#### 2.1.4 Thermostability assays

Thermostability data were obtained by using the thiol-reactive fluorophore N-[4-(7-diethylamino-4-methyl-3-coumarinyl)phenyl] maleimide (CPM), which undergoes an increase in fluorescence emission following reaction with cysteine residues [26]. A rapid procedure using a rotary qPCR machine was used, as described previously [22]. For this purpose, a 5 mg ml^− 1^ stock of CPM dissolved in DMSO was diluted 50-fold into buffer containing 20 mM HEPES/NaOH pH 8.0, 150 mM NaCl, 0.1% dodecyl-maltoside and 0.10 mg ml ^− 1^ tetraoleoyl cardiolipin (18:1), vortexed and the solution was allowed to equilibrate in the dark at room temperature for 10 min. Three micrograms of protein was added into a final volume of 45 μL buffer B, and 5 μL CPM working solution was added, and the solution was vortexed and allowed to equilibrate in the dark for 10 min at room temperature in the presence of 50 μM ADP/20 μM CATR or 50 μM ADP/20 μM BKA for the compounds to take effect, or on ice for 10 min without inhibitor to keep the protein stable. Fluorescence of the CPM dye was measured on a Qiagen Rotorgene Q using the HRM channel, which provides excitation light at 440–480 nm with emission detected at 505–515 nm. Measurements were made in 1 °C intervals from 25 to 90 °C with a ‘wait between reading’ set to 4 s, which equated to a ramp rate of 5.6 °C/min, following an initial pre-incubation step of 90 s. Data were analyzed and melting temperatures (the inflection point of the melting curve) were determined with the software supplied with the instrument. Four micrograms of protein were used in each assay. In total 27 experiments were carried out; three independent purifications, three separate Rotor-Gene-Q runs, each in triplicate.

### 2.2 Protein expression in *Lactococcus lactis*

#### 2.2.1 Construction of lactococcal wild-type and mutant AAC expression strains

For optimal expression in *Lactococcus lactis* [27], residues 2–14 and 309–315 were removed by PCR, including the His-tag, and the resulting genes were cloned into the expression vector pNZ8048 under the control of a nisin A-inducible promoter [28]. The plasmids were transformed in *L. lactis* strain NZ9000 by electroporation, as previously described [27], [29], [30]. Vectors were isolated by miniprep (Qiagen), according to the manufacturer's instructions with one alteration: 10 mg ml^-1^ lysozyme was added to the lysis buffer and the resuspended cells were incubated at 55 °C for twenty minutes prior to lysis. Gene insertions were confirmed by sequencing.

#### 2.2.2 Growth of *Lactococcus lactis* and membrane isolation

Pre-cultures of *L. lactis* were obtained by inoculating M17 medium supplemented with 1% (*w*/*v*) glucose and 5 μg ml^− 1^ chloramphenicol from glycerol stocks and incubating the cultures overnight at 30 °C with no aeration. The cells were diluted to a starting A_600_ of 0.1 in fresh M17 medium supplemented with 1% (*w*/*v*) glucose and 5 μg ml^− 1^ chloramphenicol. Cells were grown at 30 °C with no aeration until the A_600_ reached 0.5; the expression of the recombinant proteins was induced by addition of nisin A with a dilution of 1:10,000 of spent M17 medium from nisin A-excreting *L. lactis* strain NZ9700. The cells were grown for a further 2 h at 30 °C, harvested by centrifugation (6000 g, 10 min, 4 °C), resuspended in PBS buffer and collected by centrifugation as before. The cells were subsequently resuspended in 50 ml PBS buffer and disrupted mechanically with a cell disruptor (Constant Cell Disruption Systems) at 30 kpsi. Whole cells and debris were removed by centrifugation (10,800 g, 15 min, 4 °C), and the membranes were collected by ultracentrifugation (138,000 g, 1 h, 4 °C). Pellets were resuspended in PBS buffer to a total protein concentration of approximately 5 mg ml^− 1^ and stored in liquid nitrogen.

#### 2.2.3 Preparation of lipid for membrane vesicle fusions

For liposome preparation, *E. coli* polar lipid extract and egg yolk phosphatidylcholine (20 mg ml^− 1^ in chloroform) were mixed in a mass ratio of 3:1. The chloroform was evaporated under a stream of nitrogen, the lipids were resuspended in PBS buffer with a homogenizer to a final concentration of 20 mg ml^− 1^ and frozen in liquid nitrogen.

#### 2.2.4 Membrane vesicle fusions and transport assays

To make membrane fusions, 1 mg of lactococcal membranes were mixed with 5 mg liposomes, diluted to a final volume of 900 μl with PBS, and fused by seven cycles of freezing in liquid nitrogen and thawing at room temperature before storage in liquid nitrogen. The membrane vesicle fusions were thawed, and 100 μl 10 × substrate added. Vesicles were extruded 11 times through a 1-μm polycarbonate filter, passed through a pre-equilibrated PD10 column to remove external substrate, and collected in 1.6 ml PBS buffer. Transport assays were carried out by using a Hamilton MicroLab Star robot (Hamilton Robotics Ltd., Birmingham). Transport of ^14^C-labeled ADP was initiated by the addition of 100 μL PBS buffer with 1.5 μM ^14^C-ADP (2.22 GBq mmol^− 1^) to 5 μg fused membranes in a MultiScreen_HTS_-HA 96-well filter plate (pore size = 0.45 μm; Millipore). The transport was started to give 0 s, 10 s, 20 s, 30 s, 45 s, 60 s, 150 s, 5 min, 7.5 min, 10 min and 15 min incubation times with ^14^C-ADP, and stopped by the addition of 200 μl ice-cold PBS buffer and filtering using a vacuum manifold, followed by an additional wash step with 200 μl ice-cold PBS buffer. Levels of radioactivity in the vesicles were measured by the addition of 200 μl MicroScint-20 (Perkin Elmer) and by quantifying the amount of radioactivity with a TopCount scintillation counter (Perkin Elmer). Initial rates were determined from the linear part of the uptake curves in the first 60 s; background binding of ^14^C-ADP to *lactococcal* membranes was determined with an uninduced control strain, and subtracted from the rate. The specific ^14^C-ADP uptake rates (per mg AAC) were calculated following the quantification of wild type and mutant AAC by Western blot analysis, using a standard curve of purified MtAAC.

## 3 Results

The putative cytoplasmic salt bridge network of mitochondrial ADP/ATP carrier (AAC) from *Myceliophthora thermophila* [22] consists of residues D101 and K104, D205 and K208, and D299 and Q302 on transmembrane α-helices H2, H4 and H6, respectively. They are located at the water-membrane interface on the cytoplasmic side of the carrier and do not interact when the carrier is in the cytoplasmic state [21] (Fig. 1B and C). It has been proposed that in the matrix state the cytoplasmic network engages by forming bonds between residues D101-K208, D205-Q302, and D299-K104 (Fig. 1D) [16]. Previously, we have introduced a qualitative measure for the interaction energy of these networks by counting the number of potential salt bridge and hydrogen bond interactions, assuming that hydrogen bonds have about half the interaction energy of a salt bridge [16]. On this basis the cytoplasmic network of wild-type AAC has a putative interaction energy of 2.5, based on two salt bridges and one hydrogen bond (Fig. 1D). We have introduced a series of mutations into the carrier with the aim to increase or decrease the overall interaction energy of the putative cytoplasmic network. The Q302K mutation results in a three-fold symmetrical salt bridge network with an interaction energy of 3.0 (Fig. 1E). The Q302A mutation eliminates the hydrogen bond interaction, generating a network with an interaction energy of 2.0 (Fig. 1E). In this way the interaction energy of the cytoplasmic network was varied from 3.0 to 0.5 (Fig. 1E).

Since polar interactions are relatively large contributors to the overall stability of proteins, we reasoned that these mutations should affect the stability of the carrier in a state-dependent manner. To obtain a measure for protein stability, we employed a thermostability assay that uses the probe N-[4-(7-diethylamino-4-methyl-3-coumarinyl)-phenyl]-maleimide (CPM), which reacts with exposed protein thiols to form a fluorescent adduct [26]. In the procedure, the temperature of purified protein samples is increased from 25 to 90 °C while protein unfolding is monitored with CPM, as buried cysteine residues become solvent exposed due to thermal denaturation [22]. The apparent melting temperature is derived from the peak in the derivative of the melting curve, which corresponds to the temperature at which approximately half of a given protein population is unfolded (melted). For the assay, AAC was purified in dodecyl-maltoside in the presence of tetraoleoyl-cardiolipin, which is required to stabilize the unliganded carrier. This AAC has only two cysteines: C65 in matrix α-helix h12 and C229 in transmembrane α-helix H5 (Fig. 1B and C).

**Fig. 2:**
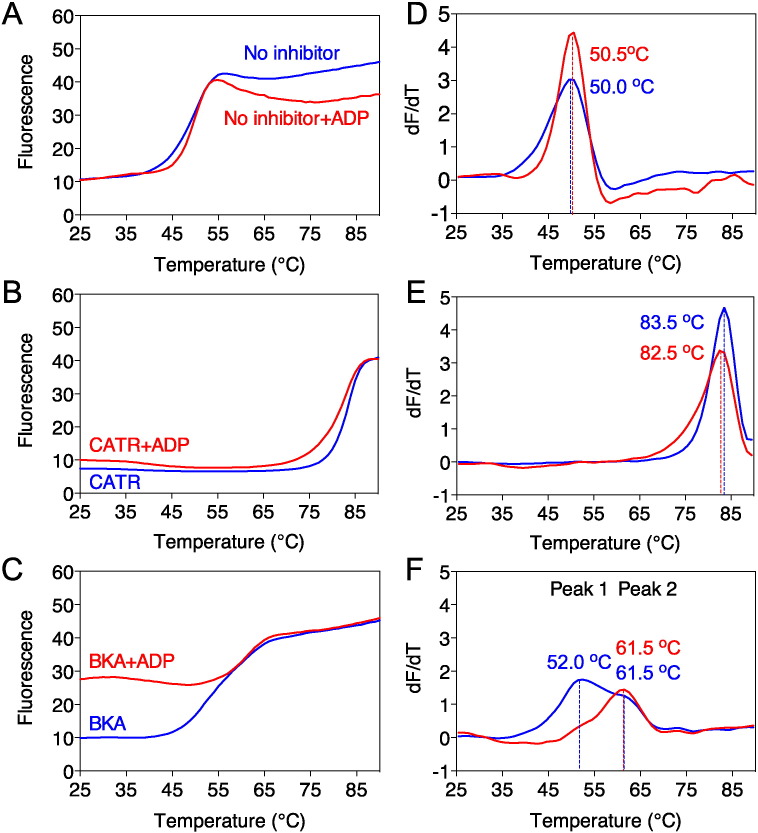
Thermostability profiles of purified wild-type AAC in the presence of inhibitors and substrates. Representative unfolding profiles of uninhibited AAC (A), CATR-inhibited AAC (B) and BKA-inhibited AAC (C) in the presence (red line) or absence (blue line) of ADP (left panels). (D–F) Derivatives of the unfolding profiles of A–C, respectively. The apparent melting temperatures are indicated.

The unfolding profiles of the wild-type AAC were determined in the absence or presence of carboxyatractyloside (CATR) or bongkrekic acid (BKA) (Fig. 2). As the binding of these inhibitors is state-dependent, it was necessary to also add the substrate ADP to allow cycling between states. Importantly, ADP binds with low affinity, which is insufficient to interfere with binding or release of these high affinity inhibitors. Consequently, even in the presence of ADP, the carrier is locked in either the aborted matrix or cytoplasmic state. In the absence of inhibitor, the carrier had a low apparent melting temperature of ~ 50 °C, whether ADP was present or not (Fig. 2A and D). Addition of CATR resulted in a ~ 33 °C increase in the apparent melting temperature of wild-type AAC, whereas the addition of ADP had little effect on protein stability, indicating that the carrier was indeed locked in an aborted state (Fig. 2B and E).

**Fig. 3:**
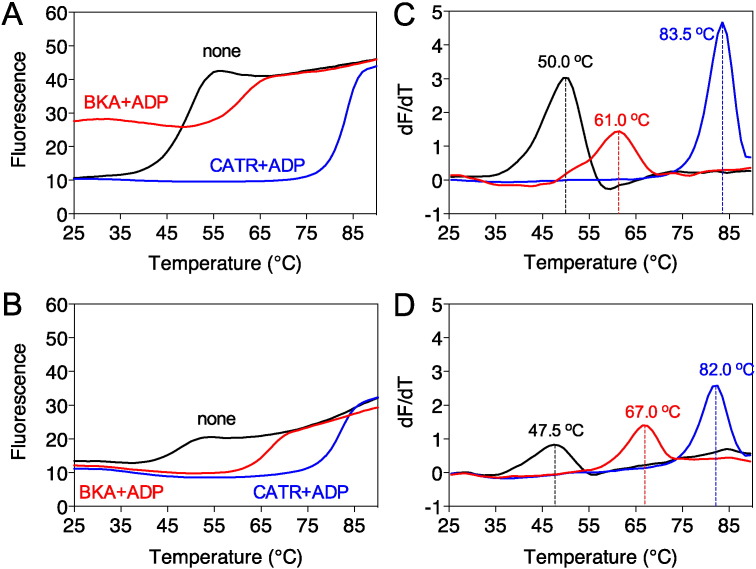
Residue C65 becomes accessible when the ADP/ATP carrier is in the matrix state. Representative unfolding profiles of purified wild-type AAC (A) or mutant C65A (B) without additions (black line) or inhibited with CATR (blue line) or BKA (red line) in the presence of ADP. (C and D) Derivatives of the unfolding profiles (A and B), respectively. The apparent melting temperatures are indicated.

BKA locks the protein in the matrix state [31], where the cytoplasmic network is predicted to form. The unfolding profile of wild-type AAC incubated with BKA alone had two apparent melting temperatures, indicating that the population was divided in two different conformational states (Fig. 2C and F). The larger population had an apparent melting temperature of 52 °C, similar to unliganded AAC (50 °C). The smaller population had an apparent melting temperature of 61 °C, indicating that the carrier is in the BKA-inhibited state, which leads to an increase in thermostability by 11 °C. The combination of ADP and BKA had two effects. First, the population was homogeneous with an apparent melting temperature of 61 °C. This result confirmed that the substrate ADP is able to shift all of the carriers to the matrix state for binding of BKA, as observed previously [4]. Second, a higher baseline of fluorescence prior to temperature ramping was observed, which corresponded to about half the total fluorescence signal. This result indicated that one of the two cysteines was already exposed when all of the carriers were locked in the matrix state. To investigate this effect further, we mutated C65 in matrix α-helix h12 of wild-type AAC to alanine (C65A) (Fig. 1B and C). The mutant C65A was subjected to thermostability assays in the presence of ADP with either CATR or BKA. The initial fluorescence levels of the uninhibited and CATR-inhibited C65A mutant were low, similar to the wild-type protein (Fig. 3A and B). However, in the presence of BKA the raised baseline fluorescence apparent in wild-type AAC was absent in the C65A mutant. The total fluorescence of the unfolded C65A mutant after denaturation was about half that of the unfolded wild-type AAC. Taken together, these results show that C65 becomes available when the carrier is locked in the matrix state by BKA, whereas C229 only becomes available as a result of thermal denaturation. Therefore, the raised baseline fluorescence associated with labeled C65 is a marker of the carrier being in the BKA-inhibited matrix state. These results also show that the unliganded AAC is in the cytoplasmic state (no raised baseline) and that ADP is required for the entire population of carriers to convert from the cytoplasmic to the matrix state for BKA binding.

The apparent melting temperatures for wild-type and mutant C65A were very similar in the absence of inhibitor (50.0 and 47.5 °C, respectively) or the presence of CATR plus ADP (83.5 and 82.0 °C, respectively) (Fig. 3C and D), demonstrating that both states are not affected by the mutation. There was a slightly bigger difference between the two in the presence of BKA plus ADP (61 and 67 °C, respectively), indicating that adduct formation at C65 prior to the thermal ramp may lead to mild destabilization of the wild-type protein in the matrix state. In summary, the unliganded AAC in detergent is able to bind the state-specific inhibitors CATR and BKA and to cycle between states through substrate-induced conformational changes, indicating that it has retained the known properties of the protein in the membrane. All subsequent experiments were carried out in the presence of ADP to obtain a homogeneous population of carrier in an aborted cytoplasmic or matrix state.

### 3.1 Interaction energy of the cytoplasmic network determines the stability of the matrix state

**Fig. 4:**
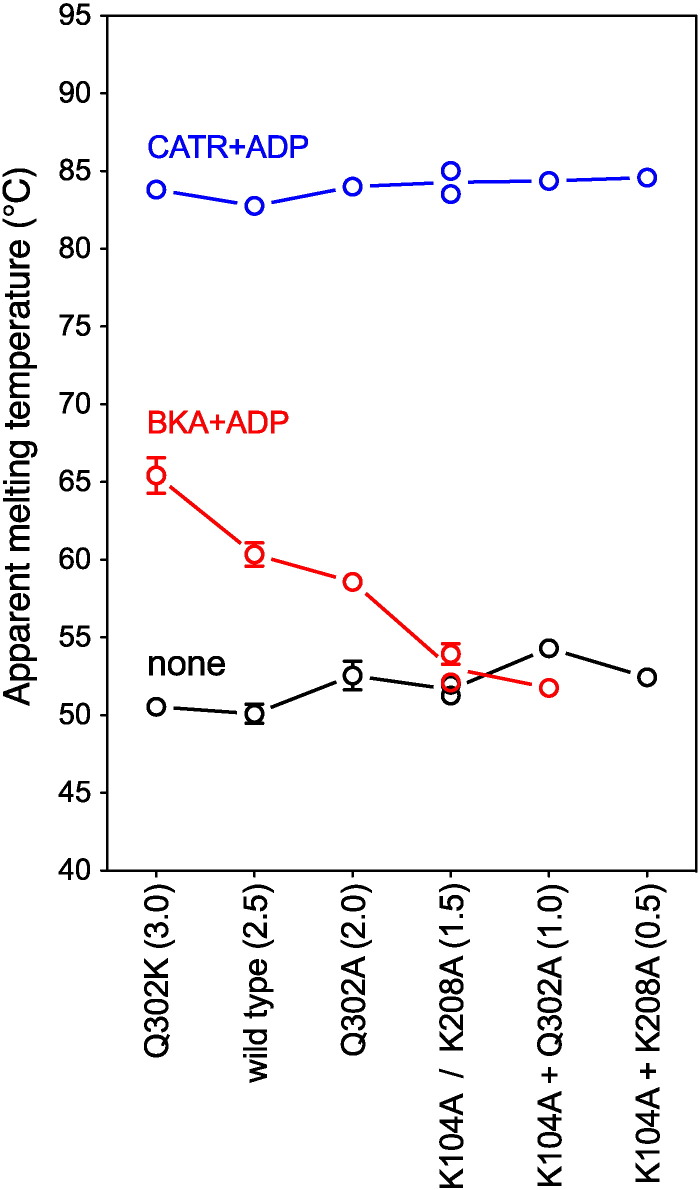
Average apparent melting temperatures of purified wild-type and mutant AAC. Apparent melting temperature of AAC was determined in the absence of inhibitor (black) or in the presence of CATR plus ADP (blue) or BKA plus ADP (red). The mutant K104A + K208A could not be locked in the BKA-inhibited matrix state (see text for details), and thus the melting temperature could not be determined. The theoretical interaction energy is shown between brackets (see legend to Fig. 1). The data are represented by the average and standard deviation of 27 experiments using three separate protein purifications, three separate Rotor-Gene-Q runs, each in triplicate. Representative unfolding profiles are shown in Fig. S1.

To test the effect of the cytoplasmic network mutations on protein stability, we determined the apparent melting temperatures for the wild-type and all mutant AAC (Fig. 1D and E) in the absence or presence of CATR + ADP or BKA + ADP (Fig. 4 and Fig. S1). In the absence of inhibitor, the melting temperatures of the wild type and mutants were very similar (50–55 °C). When inhibited with CATR + ADP, the melting temperatures of *all of* the mutants increased by ~ 30 °C (83–85 °C). These results show that the mutant proteins are well folded, as they are still capable of binding CATR in the cytoplasmic state.

When the wild-type and mutant carriers were incubated with BKA + ADP, there was a strong correlation between the apparent melting temperatures and the putative interaction energies of the cytoplasmic network. Relative to wild-type AAC, the melting temperature of Q302K was 5 °C higher, indicating that the mutant was more stable in the matrix state, consistent with a higher interaction energy. The apparent melting temperature of Q302A with a putative interaction energy of 2.0 was 58.6 °C, almost two degrees lower than wild type. The melting temperatures of K104A and K208A with interaction energies of 1.5 were 4.7 and 6.5 °C lower than Q302A, respectively. The mutant K104A + Q302A with a cytoplasmic network interaction energy of 1.0 had a melting temperature of 51.8 °C, the lowest that could be determined. The mutant with the weakest network, K104A + K208A, could not be locked in the matrix state by BKA + ADP, as was evident from the absence of a raised baseline fluorescence (Fig. S1).

### 3.2 Effect of the cytoplasmic network on kinetic parameters of transport

**Fig. 5:**
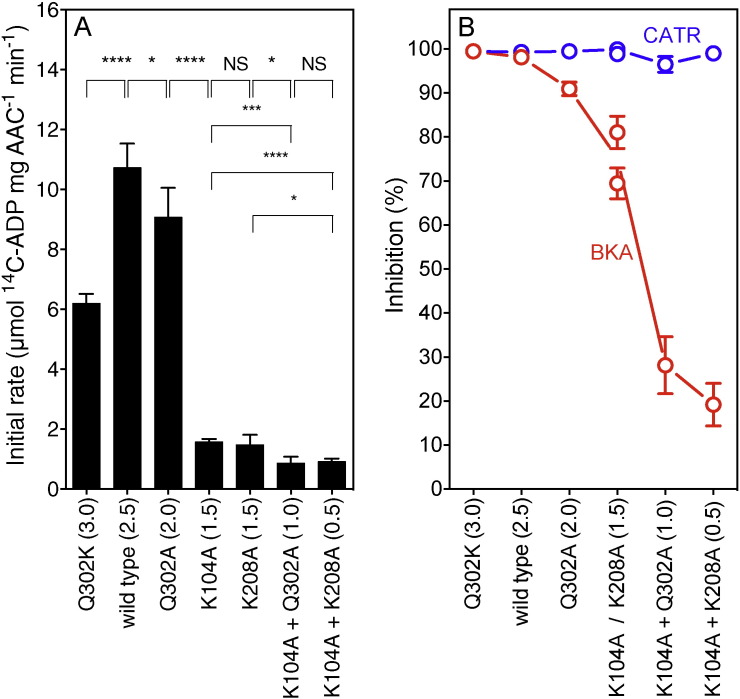
Specific initial transport rates and percentage inhibition of the wild-type and mutant AAC. The specific initial transport rates were determined with fused membranes of *Lactococcus lactis* expressing the wild-type or mutant carriers (A), loaded with ADP. The theoretical interaction energy is shown between brackets (see legend to Fig. 1). Percentage of inhibition of transport (B) in the presence of CATR (blue line) or BKA (red line). The specific ADP uptake rates (per mg of AAC) were corrected for background binding. The error bars represent the standard deviation of four assays. Student t-tests: P > 0.05, not significant (NS); P > 0.01, *; P > 0.001, **; P > 0.0001, ***; P < 0.0001, ****.

We investigated the effect of the mutations of the cytoplasmic network on the transport rate in fused membrane vesicles of *Lactococcus lactis* [29] by monitoring the specific initial rate of exchange of internal unlabeled ADP for external labeled ^14^C-ADP (Fig. 5 and Fig. S2). Controls using membrane-impermeable CATR showed that all of the carriers were oriented with the cytoplasmic side to the outside. The transport experiments showed that all mutants transported ADP, albeit with different rates, indicating that they were well folded consistent with the effect of the CATR on protein stability (Fig. 4). The specific initial transport rate was the highest for the wild-type AAC (10.7 ADP μmol mg^− 1^ min^− 1^, network 2.5). The mutants Q302K (6.2 μmol mg^− 1^ min^− 1^, network 3.0) and Q302A (9.1 μmol mg^− 1^ min^− 1^, network 2.0) transported ADP at slightly lower rates. However, mutants with cytoplasmic network interaction energies of 1.5 or lower had much lower transport rates (Fig. 5A). K104A + K208A (network 0.5) had a transport rate of 0.9 μmol mg^− 1^ min^− 1^, about twelve times less than that of wild-type AAC.

We also determined the effect of the cytoplasmic network mutations on the apparent K_m_ and V_max_ of transport (Fig. S3). The K_m_ values were in the range of 8.7–12.4 μM for the wild type and most of the mutants (Table 1), indicating that the mutations did not affect substrate binding significantly. The only exception was Q302K, which had a lower apparent K_m_ of 2.2 μM. The V_max_ values were high for the wild-type and Q302A mutant, being 65.7 and 73.0 μmol ADP mg^− 1^ min^-1^, respectively. The V_max_ value of Q302K (network 3.0) was approximately one third of the wild-type AAC, whereas the other V_max_ values decreased with decreasing interaction energy of the cytoplasmic network (Table 1). These results show that interactions of the cytoplasmic network are important determinants of the transport rate of AAC. The effect of the inhibitors on the transport rate was also determined. The results show that addition of CATR led to a full inhibition of transport by the wild-type and mutant AAC (Fig. 5B and S2). In contrast, the inhibition by BKA was dependent on the interaction energy of the cytoplasmic network. Mutant Q302K and wild-type AAC were almost completely inhibited by BKA (> 98%), whereas mutant Q302A was inhibited to 91%, K104A to 81%, K208A to 69%, K104A + Q302A to 28%, and K104A + K208A to 19%.

## 4 Discussion

We have previously proposed a domain-based alternating access mechanism for mitochondrial carriers [16], [21]. Key to this mechanism is the formation of the cytoplasmic salt bridge network when the carrier is in the matrix state, which is demonstrated experimentally in this work. A series of mutant carriers were engineered in which the interaction energy of the cytoplasmic network increased or decreased compared to the wild-type. All of these mutants were capable of transporting ADP (Fig. 5A), albeit with different rates, and were fully inhibited by CATR (Fig. 5B). In the CATR-inhibited state, which is a locked cytoplasmic state, the apparent melting temperature was very similar for all of the network mutants, approximately 83 °C, confirming that they all were able to bind CATR (Fig. 4). The marked increase in thermostability due to CATR binding is consistent with the structures of bovine AAC1 [13] and yeast Aac2p and Aac3p [21], which show that CATR is bound to the central cavity by an extensive network of salt bridges, hydrogen bonds and hydrophobic contacts, cross-linking most of the transmembrane α-helices. The observations that thermostability increases by the same amount for all mutants also shows that the residues of the cytoplasmic network are not interacting in the aborted cytoplasmic state in agreement with the structures [13], [21]. The initial fluorescence of the unfolding curves for the uninhibited carriers was very low, demonstrating that all carriers were well folded and in the cytoplasmic state at the start of the thermal stability assays (Fig. S1). Strikingly, when the carriers were locked in the matrix state by BKA by the addition of ADP, a strong correlation between the predicted interaction energies of the cytoplasmic network and the apparent melting temperatures was observed (Fig. 4). Notably, the thermal stability of the Q302K mutant was higher than that of the wild-type in agreement with a replacement of a hydrogen bond with a salt bridge. All other mutations led to a decreased stability in the BKA-inhibited state, correlating strongly with the expected interaction energy. With the exception of K104A + K208A, all of the mutants and the wild type could be locked in the matrix state by BKA, as they exhibited an increased baseline of fluorescence, indicative of C65 being accessible to the probe. It has been observed previously that sulfhydryl reagents react with cysteine residues in the matrix α-helices, which leads to inhibition of the transport activity [32]. Taken together, the results demonstrate that residues of the cytoplasmic network are key determinants of protein stability only when the carriers are locked in the matrix state, which is consistent with formation of the cytoplasmic network. The strict correlation also indicates that the network forms symmetrically, as all of interactions of the network were shown to be important even though their positions differed. In further agreement, the mutants K104A and K208A (both with an interaction energy of 1.5) had similar thermostabilities and transport properties, even though different bonds in the network were affected by the mutations. These thermostability results further demonstrate that individual bonds between amino acid residues can be detected in a state-dependent manner, which is a useful tool for the characterization of detergent-solubilized membrane proteins.

Another important observation is that the cytoplasmic network is a key determinant of the inhibitory effect of BKA (Fig. 5B), providing further support for the notion that formation of the matrix state and binding of BKA are interlinked, as noted previously [4]. BKA is a relatively long and flexible molecule with only three carboxylic groups, providing fewer and weaker binding opportunities than CATR (Fig. S4). In agreement, the shift in apparent melting temperature is much smaller (11 °C for BKA *versus* 33 °C for CATR for the wild-type). Thus the formation of the cytoplasmic network is required for BKA to exert its inhibitory effect. These results demonstrate also that the thermostability assays are useful for characterizing ligand-protein interactions, as shown previously for GDP and cardiolipin binding to the uncoupling protein [33].

**Fig. 6:**
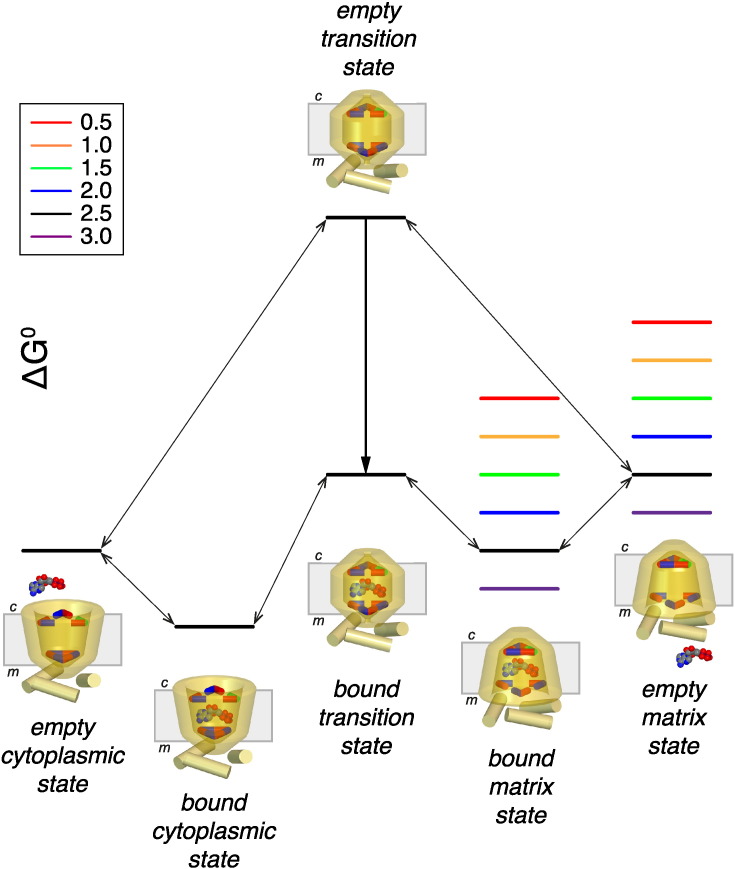
Schematic energy diagrams of the wild-type and mutant fungal ADP/ATP carriers with different interaction energies of the cytoplasmic network. The energy states of the wild-type carrier are shown in black, whereas those of the mutant carriers are shown in rainbow colors, varying from an putative interaction energy of the cytoplasmic salt bridge network of 3.0 (purple) to 0.5 (red), as indicated in the legend. Arrows indicate the reversible steps between transport states of the wild type. The bold arrow indicates the change in free energy upon substrate binding in the transition state. Pictorial representations of the transport states are also shown. The transition state is an occluded state with the residues of the networks not or only weakly interacting. The mitochondrial matrix (m) and the intermembrane space, which is continuous with the cytoplasm (c) are also indicated.

The effects of the mutations on the transport cycle can be explained with a schematic energy diagram of the transport states (Fig. 6). The Induced Transition Fit theory of transport catalysis, formulated by Klingenberg, has introduced the important concept that the energy barrier for conversion between states is lowered by substrate binding [34], [35]. At the time, however, the energy barriers of the transport process were not structurally defined for mitochondrial carriers. We now know that carriers operate as monomers [23], [32], [36] with a single substrate binding site in the central cavity flanked by two salt bridge networks [13], [14], [16], which are the largest contributors to inter-domain interactions [21].

The transition state in the absence of substrate is a dynamic short-lived high-energy state in which the residues of the salt bridge networks are not or only weakly interacting (Fig. 6, top). The formation of salt bridge networks in the substrate-free matrix or cytoplasmic state lowers the energy levels considerably (Fig. 6). Therefore, there is a very low probability for the networks to disrupt spontaneously and for the carrier to reach the high energy levels of the transition state due to thermal energy. Consequently, the carrier is trapped in the low energy levels of the matrix or cytosolic state in agreement with the experimental observations that the inter-conversion between states in the absence of substrate is extremely slow [4]. The salt bridge interactions of the matrix network are highly conserved [15], [37] and braced by glutamine residues to increase the overall interaction energy of the network to 3.5 in the qualitative measure introduced earlier [16], [21].

Binding of substrate lowers the free energy depending on the strength of the bonds formed, which in turn depends on the precise geometry of the substrate in its binding site (lower black lines, Fig. 6). The free energy of the transition state is thus lowered sufficiently for the substrate-bound carrier to cycle between the matrix and cytosolic states in both directions by thermal energy [34], [35]. The same principles are likely to apply to the mechanism of other transport proteins, but the differences in energy levels may be larger in the case of mitochondrial ADP/ATP carriers, because of the salt bridge interactions of the networks. Consequently, a substantial amount of interaction energy must be involved in substrate binding too in order to lower the energy barrier sufficiently for transport to occur. The substrate binding site of the bovine mitochondrial ADP/ATP carrier has been proposed to consist of G182, I183 and Y186 for binding of the adenine moiety and R79, K22 and R279 for binding of the phosphate groups [14], [15], [16], [17], [18], which could form an aromatic stacking and three ionic interactions with ADP, consistent with this idea.

By introducing mutations it was possible to *increase* or *decrease* the interaction energy of the cytoplasmic network in a systematic way (colored levels, Fig. 6). Therefore, the free energy of the matrix state is altered, whereas the free energy of the cytoplasmic state is unaffected (Fig. 6). On the basis of sequence information the cytoplasmic network of the fungal ADP/ATP carriers has an interaction energy of 2.5, whereas mammalian ones have an interaction energy of 3.0. Since all of the wild-type and mutant carriers lack a raised baseline fluorescence in the absence of ADP and BKA (Fig. S1), they are most likely in the cytoplasmic state, indicating that the substrate-free cytoplasmic state has a lower energy level than the substrate-free matrix state, as depicted in Fig. 6.

The effect of the mutations on the kinetic parameters of transport were also determined (Table 1). There was a marked effect on the Vmax of transport caused by mutations of the cytoplasmic network. The Vmax values were high when the interaction energies of the cytoplasmic network were in the range of 3.0–2.0, but very low in the range of 1.5–0.5. The energy diagram can explain why there is a sharp decline in the transport rate, as the probability of forming matrix states will be very low in the latter mutants, affecting the overall transport rate (Fig. 6). The energy levels of the substrate-free matrix state of the mutant with weak networks could be close to the energy level of the substrate-free or empty transition state. Thus they could convert spontaneously to the cytoplasmic state in the absence of substrate, but they would be trapped there, because the conversion back to the matrix state represents a large change in free energy. In agreement the mutant with the weakest cytoplasmic network was trapped in the cytoplasmic state (see lack of raised fluorescent baseline in the presence of BKA and ADP, Fig. S1).

The apparent Km of transport for wild-type AAC was 9.3 μM, which is compatible with values for the ADP/ATP carrier of *Saccharomyces cerevisiae* [38]. The Km values of most mutants were not significantly different from that of the wild type, consistent with the location of residues in the cytoplasmic network away from central substrate binding site, where they are unlikely to have a direct involvement in substrate binding [14], [15]. In the case of the Q302K mutant, the Km value was much lower, 2.2 μM. This mutation lowers the energy level of the matrix state involved in substrate binding (Fig. 6) and thus the probability of being in that state would be much higher. Consequently, the Km would be lower, as it represents the apparent dissociation constant for the sum of all bound states, including the substrate bound matrix state [39]. With these data we are building a statistical mechanics model to test these and other properties of the mitochondrial ADP/ATP carrier.

In conclusion, the experimental data in this paper support a unique transport mechanism that provides an alternating access of the substrate to the binding site by the simultaneous rotation of the three domains around a central translocation pathway through the disruption and formation of two salt bridge networks.

## Author contributions

M.S.K. and E.R.S.K. designed research; M.S.K. and M.K carried out the mutagenesis, thermostability assays and transport assays, following advice of P.G.C. and E.R.S.K; M.S.K., P.G.C., R.S. and E.R.S.K. analyzed data; and M.S.K., P.G.C., R.S. and E.R.S.K. wrote the paper.

## Transparency document

Transparency document

## Figures and Tables

**Table 1:** Kinetic parameters determined for wild-type and mutant MtAAC.

Mutant	Apparent Km (μM)⁎	Vmax (μmol ^14^C-ADP mg AAC^− 1^ min^− 1^)⁎
Q302K	2.2 ± 0.3	21.9 ± 0.9
Wildtype	9.3 ± 1.8	65.7 ± 7.1
Q302A	11.2 ± 2.3	73.0 ± 9.0
K104A	12.4 ± 3.7	19.0 ± 3.5
K208A	9.4 ± 2.0	14.8 ± 1.8
K104A + Q302A	11.9 ± 4.0	11.6 ± 2.3
K104A + K208A	8.7 ± 1.7	3.9 ± 0.5

⁎ Values represent the average and standard error of four measurements.
