# Corrigendum to “Formation of a cytoplasmic salt bridge network in the matrix state is a fundamental step in the transport mechanism of the mitochondrial ADP/ATP carrier” [Biochim. Biophys. Acta 1857 (2016) 14–22]

**DOI:** 10.1016/j.bbabio.2016.09.013

**Published:** 2016-12

**Authors:** Martin S. King, Matthew Kerr, Paul G. Crichton, Roger Springett, Edmund R.S. Kunji

**Affiliations:** Medical Research Council Mitochondrial Biology Unit, Cambridge Biomedical Campus, Wellcome Trust/MRC Building, Hills Road, Cambridge CB2 0XY, UK

The labeling of Fig. 1 panel B and C is incorrect, as residues D101 and K104 have been swapped with residues D205 and K208, respectively. Consequently, the fourth line on page 17 is also incorrect and should read: It has been proposed that in the matrix state the cytoplasmic network engages by forming bonds between residues D101-Q302, D205-K104, and D299-K208.

The authors would like to apologize for any inconvenience caused.

