# Supplementary material for: Formation of a cytoplasmic salt bridge network in the matrix state is a fundamental step in the transport mechanism of the mitochondrial ADP/ATP carrier
Source: Biochim Biophys Acta. 2016 Jan;1857(1):14–22. doi: 10.1016/j.bbabio.2015.09.013 (PMC4674015; doi:10.1016/j.bbabio.2015.09.013)
Supplement: Supplementary file 2 — Supplementary figures [file mmc2.docx]

**Supplementary Figures and Legends**

**Formation of a cytoplasmic salt bridge network in the matrix state is a fundamental step in the transport mechanism of the mitochondrial ADP/ATP carrier**

Martin S. King, Matthew Kerr, Paul G. Crichton, Roger Springett and Edmund R.S. Kunji*

Medical Research Council Mitochondrial Biology Unit, Wellcome Trust / MRC Building, Hills Road, Cambridge, CB2 0XY, UK.


­

Figure S1: Typical unfolding profiles of the cytoplasmic network mutants in the absence of inhibitor (black trace), in the presence of 50 μM ADP and 20 μM carboxyatractyloside (blue trace) or 50 μM ADP and 20 μM bongkrekic acid (red trace). Four micrograms of protein was used in each assay. In total 27 experiments were carried out; three independent purifications, three separate Rotor-Gene-Q runs, each in triplicate.

Figure S2: Transport rates of the cytoplasmic network mutants. Vesicles of *Lactococcus lactis* membranes expressing the carriers, loaded with 5 mM ADP, were assayed without inhibitor (black trace), or in the presence of 20 μM carboxyatractyloside (blue trace) or 20 μM bongkrekic acid (red trace). Transport was initiated with the external addition of 1.5 μM ^14^C-ADP. To obtain specific transport rates, AAC expression levels were quantified and the rate was corrected for background binding of ADP. The data are represented by the average and standard deviation (n = 4).

Figure S3: Determination of Km and Vmax for the cytoplasmic network mutants. Vesicles of *Lactococcus lactis* membranes expressing the carriers, loaded with 5 mM ADP. Transport was initiated with the external addition of either 0.01 μM, (filled circle), 0.05 μM, (filled square), 0.10 μM, (filled triangle), 0.50 μM, (cross), 1 μM, (open circle), 5 μM, (open square) or 10 μM ^14^C-ADP, (open triangle). The bottom right graph shows initial rate plotted against ADP concentration; Q302K; filled circle, MtAAC; filled square, Q302A; filled triangle, K104A; cross, K208A; open circle, K104A+Q302A; open square, K104A+K208A; open triangle. To obtain specific transport rates, AAC expression levels were quantified and the rate was corrected for background binding of ADP.The data are represented by the average and standard deviation (n = 4).


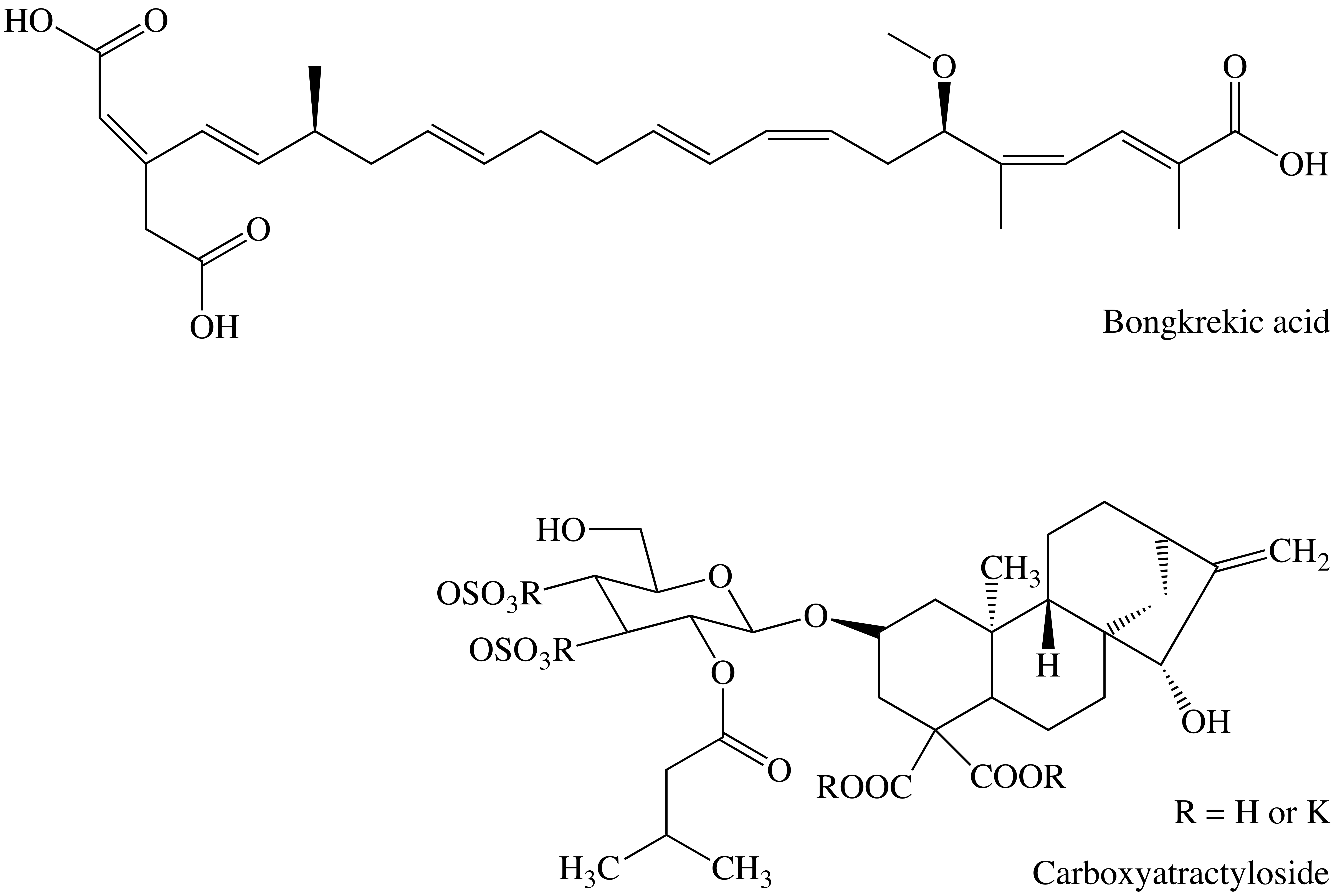


Figure S4: The chemical structures of carboxyatractyloside and bongkrekic acid
